# Prenatal Exposure to Bisphenol A and Child Wheeze from Birth to 3 Years of Age

**DOI:** 10.1289/ehp.1104175

**Published:** 2012-02-14

**Authors:** Adam J. Spanier, Robert S. Kahn, Allen R. Kunselman, Richard Hornung, Yingying Xu, Antonia M. Calafat, Bruce P. Lanphear

**Affiliations:** 1 Department of Pediatrics, and; 2 Department of Public Health Sciences, Penn State University Hershey Medical Center, Hershey, Pennsylvania, USA; 3 Department of Pediatrics, Cincinnati Children’s Hospital Medical Center, Cincinnati, Ohio, USA; 4 Division of Laboratory Sciences, National Center for Environmental Health, Centers for Disease Control and Prevention, Atlanta, Georgia, USA; 5 Child and Family Research Institute, BC Children’s Hospital and Faculty of Health Sciences, Simon Fraser University, Vancouver, Canada

**Keywords:** bisphenol A, BPA, child, cotinine, prenatal, tobacco, wheeze

## Abstract

Background: Bisphenol A (BPA), an endocrine-disrupting chemical that is routinely detected in > 90% of Americans, promotes experimental asthma in mice. The association of prenatal BPA exposure and wheeze has not been evaluated in humans.

Objective: We examined the relationship between prenatal BPA exposure and wheeze in early childhood.

Methods: We measured BPA concentrations in serial maternal urine samples from a prospective birth cohort of 398 mother–infant pairs and assessed parent-reported child wheeze every 6 months for 3 years. We used generalized estimating equations with a logit link to evaluate the association of prenatal urinary BPA concentration with the dichotomous outcome wheeze (wheeze over the previous 6 months).

Results: Data were available for 365 children; BPA was detected in 99% of maternal urine samples during pregnancy. In multivariable analysis, a one-unit increase in log-transformed creatinine-standardized mean prenatal urinary BPA concentration was not significantly associated with child wheeze from birth to 3 years of age, but there was an interaction of BPA concentration with time (*p* = 0.003). Mean prenatal BPA above versus below the median was positively associated with wheeze at 6 months of age [adjusted odds ratio (AOR) = 2.3; 95% confidence interval (CI): 1.3, 4.1] but not at 3 years (AOR = 0.6; 95% CI: 0.3, 1.1). In secondary analyses evaluating associations of each prenatal BPA concentration separately, urinary BPA concentrations measured at 16 weeks gestation were associated with wheeze (AOR = 1.2; 95% CI: 1.0, 1.5), but BPA concentrations at 26 weeks of gestation or at birth were not.

Conclusions: Mean prenatal BPA was associated with increased odds of wheeze in early life, and the effect diminished over time. Evaluating exposure at each prenatal time point demonstrated an association between wheeze from 6 months to 3 years and log-transformed BPA concentration at 16 weeks gestation only.

Asthma is one of the most common chronic and disabling conditions of childhood (Bloom et al. 2010). The prevalence of asthma in children has risen in the last few decades, currently affecting 1 in 10 children (Akinbami et al. 2011; Bloom et al. 2010). Several environmental risk factors for asthma have been identified, such as tobacco smoke and airborne pollutants (McConnell et al. 2010; Oberg et al. 2011). The reasons for the increase in childhood asthma are largely unknown, but other environmental exposures may play a role.

Recent studies have implicated exposure to plastic products in the development of childhood respiratory problems. Investigators have found an association of exposure to plastic wall materials (any plastic wall surfaces) with the development of bronchial obstruction, persistent wheeze, cough, and phlegm in children (Jaakkola et al. 1999, 2000). Jaakkola et al. (2006) found that exposure to plastic wall materials also was associated with more than two times the odds of asthma in adults. Indoor concentrations of common organic plasticizers (2,2,4-trimethyl-1,3-pentanediol monoisobutyrate and 2,2,4-trimethyl-1,3-pentanediol diisobutyrate) in schools also have been associated with increased prevalence of asthmatic symptoms in students (Kim et al. 2007). Thus, the evidence for an association between plastic exposures and wheeze is growing, but it is unclear whether specific plastic constituents, such as bisphenol A (BPA) or phthalates, act as risk factors for childhood respiratory problems.

BPA is a high-production-volume chemical used in the manufacture of some plastics and epoxy resins that are found in many consumer products. The primary route of human exposure to BPA is through dietary ingestion (Wilson et al. 2007). Unfortunately, there is little information about the variability and routes of BPA exposure in pregnant women (Braun et al. 2011). BPA was detected in the urine of > 90% of the National Health and Nutrition Examination Survey (NHANES) 2003–2004 participants > 6 years of age (Calafat et al. 2008). Perinatal BPA exposure has been reported to promote experimental asthma in mice, but this relationship has not been evaluated in humans (Midoro-Horiuti et al. 2010). The objective of this analysis was to examine the relationship between prenatal BPA exposure and wheeze in early childhood.

## Methods

We used data from the Health Outcomes and Measures of the Environment (HOME) Study for this project (Braun et al. 2009; Phelan et al. 2009). The HOME Study is a prospective birth cohort with the primary goal of investigating the effects of exposure to environmental toxicants on child growth, neurobehavioral development, and respiratory health. Between March 2003 and January 2006, the HOME Study enrolled English-speaking women at a mean ± SD of approximately 16 ± 2 weeks gestation who were ≥ 18 years and lived in a home built before 1979. We tracked the women through pregnancy and are continuing to follow their children through 5 years of age. Women resided within five Ohio counties surrounding Cincinnati, received prenatal care from one of nine participating obstetrical clinics, and delivered at one of three participating hospitals. The obstetric clinics provided us with lists of new patients, and we prescreened women for eligibility criteria. We sent study information to eligible women and placed a follow up phone call to assess interest in the study and complete screening. Approximately 37% (468 of 1,263) of eligible women agreed to participate and provided written informed consent. We excluded potential participants if they were HIV positive, taking antiepileptic medication, or were diabetic (not gestational) or had bipolar disorder or schizophrenia. Infants of participating mothers were eligible for the longitudinal study. The longitudinal study included an embedded randomized control trial of a lead hazard reduction intervention and injury hazard reduction control.

We included only children with prenatal urinary BPA concentration and respiratory outcome data for this study. These data were available for 365 children (92% of HOME Study participants: 398 live-born infants, randomly excluding one child for twin births). The institutional review boards of Cincinnati Children’s Hospital Medical Center, the Centers for Disease Control and Prevention (CDC), and the involved birth hospitals approved the HOME Study and this project.

*Exposure assessment.* We assessed prenatal BPA exposure by measuring the concentrations of BPA in serial maternal spot urine samples. We collected maternal urine and serum at enrollment (15.9 ± 1.9 weeks gestation), 26 weeks gestation, and birth. Urine and serum samples were sent to the Division of Laboratory Sciences at the CDC for analysis. Urinary BPA concentrations were quantified using online solid-phase extraction coupled with high-performance liquid chromatography/isotope-dilution tandem mass spectrometry (Ye et al. 2005a, 2005b). The limit of detection (LOD) was 0.4 μg/L. When BPA concentrations were below the LOD (< 13% of samples), we replaced these values with the LOD divided by _√_^–^2 (Hornung and Reed 1990).

We assessed tobacco exposure using serum concentrations of cotinine, a metabolite of nicotine. We used serum cotinine, a biomarker of tobacco exposure, as a covariate in analyses because of the established association of prenatal tobacco exposure with wheeze in childhood (Sherriff et al. 2001; Spanier et al. 2011). Analyses of serum for cotinine were performed using high-performance liquid chromatography/atmospheric-pressure chemical ionization tandem mass spectrometry (Bernert et al. 1997, 2000). The LOD was 0.015 ng/mL. When serum cotinine values were below the LOD (~ 35% of samples), we imputed values by sampling randomly from the left tail of a lognormal distribution. We collected other environmental exposure information (i.e., pet ownership, cockroach exposure, and house characteristics) by survey as described below.

*Outcome measure.* After birth, we employed trained research assistants to survey parents every 6 months through the child’s age 3 years to collect study data. The baseline, 12-, 24-, and 36-month surveys were conducted via home visits, and the 6-, 18-, and 30-month surveys were conducted over the phone. We designed these survey questions to parallel the NHANES wheeze question (National Center for Health Statistics 1994). We asked “Has [child’s name] had wheezing or whistling in his/her chest in the last 6 months?” We also asked about the number of wheeze attacks, and we dichotomized the number of wheeze attacks at each time point (no wheeze vs. any wheeze) to minimize effects of extreme values. We used this dichotomized value as our outcome measure. We also asked if wheezing usually occurred with or followed a cold or viral illness.

*Covariates.* Trained research assistants conducted extensive surveys at baseline and every 6 months after the child was born to collect data on potential covariates, including demographic characteristics, socioeconomic status, and health status. Demographic and socioeconomic characteristics such as maternal education, race/ethnicity, occupation, income, housing volume, and health insurance status were considered as possible covariates in all models. We also considered other factors potentially associated with wheeze by evaluating them as possible covariates [e.g., prenatal tobacco exposure (cotinine), season, history and duration of breast-feeding, family history of asthma, family history of allergy, child eczema, child allergy, neonatal characteristics, pet ownership, and cockroach exposure] (Ball et al. 2000; Fleming et al. 1987; Gold et al. 1999; Oddy et al. 2002; Simoes 2003; Taussig et al. 2003; Young et al. 2000).

We categorized residence location/type into three categories based on census tract information. Family residence was categorized as urban if their census tract was located in a city defined as a metropolitan area by U.S. Census 2000 (Cincinnati, OH; Newport, KY; and Covington, KY) (unpublished data), rural if their census tract had a 2000 Census population density < 500 persons/square mile, and suburban if their census tract did not fit into urban or rural categories.

*Statistical analysis.* We first calculated descriptive statistics for all demographic, exposure, and outcome data. We used a natural log transformation for all BPA data because urinary BPA concentrations were approximately log-normal, and to minimize the influence of outlying values. We used the geometric mean and 95% confidence interval (CI) as the primary descriptors of central tendency and dispersion for BPA data.

We used the mean of creatinine standardized prenatal urinary BPA concentrations as our primary independent variable to approximate exposure over pregnancy. To calculate this variable, we first standardized BPA concentrations for urinary creatinine (micrograms of BPA per gram of creatinine) at each time point. We then calculated the mean of all available standardized BPA concentrations and log transformed the mean of prenatal BPA concentrations for each participant. We analyzed the association of prenatal urinary BPA concentration with the dichotomous outcome variable wheeze (wheeze over the previous 6 months) using generalized estimating equations (GEEs) with a logit link. GEE is an extension of logistic regression that accounts for within-subject correlation resulting from repeated outcome measurements in longitudinal studies. Therefore, all data collected during the six time points (months 6, 12, 18, 24, 30, and 36) were used in the analysis. Interpretation of the association between the exposure variable and outcome (i.e., wheeze) using odds ratios (ORs) and 95% CIs from GEEs is similar to ordinary logistic regression but represents the association with wheeze over the entire 3-year period rather than at a single point in time. We first conducted bivariate analyses to evaluate the association of prenatal BPA concentration and potential covariates with wheeze. After bivariate analysis, we conducted a multivariable analysis using forward selection techniques. In the initial multivariable analysis, we included covariates that predicted wheeze with a *p*-value ≤ 0.2 in bivariate analysis. Covariates were retained if they were significantly associated with wheeze (*p* < 0.05) or if their addition caused a > 10% change in the estimate of the association between wheeze and prenatal BPA. In all analyses (including the bivariate analyses), we included a variable for survey time point (6, 12, 18, 24, 30, or 36 months) and a variable for intervention arm to account for any potential design effects of the embedded randomized trial. We also adjusted for log-transformed maternal serum cotinine (continuous) because previous studies have demonstrated an association of cotinine with wheeze (Spanier et al. 2011). After conducting the multivariable analysis, we tested for potential multiplicative interactions of prenatal BPA concentration with all covariates on the logit scale.

We conducted secondary analyses to explore potential windows of vulnerability by replacing the mean prenatal urinary BPA concentration variable with BPA concentration measured at each of the prenatal time points (i.e., 16 weeks, 26 weeks, and at birth) in three separate analyses, each standardized for same time urinary creatinine. GEE analyses were used for these secondary analyses. SAS (version 9.2; SAS Institute Inc., Cary, NC, USA) was used for all data analyses.

## Results

**Figure 1:**
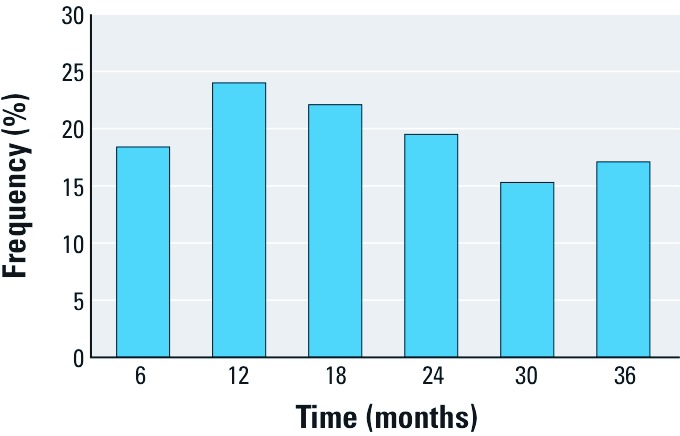
Percentage of children with wheeze over previous 6 months in the HOME Study (*n* = 365).

More than half of the child participants were female (55.6%), most had a white mother (65.5%), and most were born full term (Table 1). Compared with the 33 participants without outcome data, those for whom outcome data were available were more likely to be white, have a mother with more than a high school education, live in nonurban environment, have married parents, have an older mother, and have a higher household income. Of 365 children with maternal prenatal BPA concentration and respiratory outcome data available, 99% were born to a mother with detectable urinary BPA concentrations at some point during pregnancy. The geometric mean of the mean prenatal urinary BPA concentration was 2.4 μg/g creatinine (95% CI: 2.3, 2.6 μg/g creatinine) (Table 2). During the study, the frequency of children with wheeze during a 6-month period ranged from a low of 15.3% to a high of 24% (Figure 1). Almost all wheezing episodes occurred in relation to a cold; the frequency of children who wheezed without a cold during any 6-month period ranged from a low of 0% to a high of 2.7% (data not shown).

**Table 1:** Characteristics of study participants with available prenatal BPA concentration and wheeze data [n (%)].

Characteristic	Included	Excluded	p-Valuea
No. of participants		365 (91.7)		33 (8.3)		Not applicable
Male		162 (44.4)		21 (63.6)		0.03
Maternal race						
White		239 (65.5)		5 (15.1)		
Black		103 (28.2)		18 (54.6)		< 0.0001
Other		23 (6.3)		4 (12.1)		
Unknown				6 (18.2)		
Maternal education						
< High school		77 (21.1)		18 (54.5)		< 0.0001
≥ Any college		288 (78.9)		9 (27.3)		
Unknown				6 (18.2)		
Maternal marital status						
Married		249 (68.2)		7 (21.2)		< 0.0001
Not married		116 (31.8)		20 (60.6)		
Unknown				6 (18.2)		
Household income						
< $25,000		87 (23.8)		15 (45.4)		
$25,000–50,000		78 (21.4)		6 (18.2)		0.003
$50,000–80,000		91 (24.9)		1 (3.0)		
> $80,000		102 (27.9)		3 (9.1)		
Unknown				8 (24.2)		
Residence						
Rural		12 (3.3)		1 (3.0)		
Suburban		133 (36.4)		6 (18.2)		0.04
Urban		220 (60.3)		26 (78.8)		
History of maternal allergy		157 (43.0)		11 (40.7)		0.82
Prenatal serum cotinine concentration, μg/L [geometric mean (95% CI)]		0.06 (0.04, 0.07)		1.15 (0.41, 3.23)		< 0.0001
Gestational age, weeks (mean ± SD)		39.0 ± 1.8		38.0 ± 2.8		0.07
aRepresents a test (chi-square or t-test) of the difference between the children in the included and excluded groups.						

**Table 2:** Prenatal creatinine-standardized urinary BPA concentrations (μg/g).

Collection time	n	Geometric mean (95% CI)	Percent > LODa
16 weeks gestation		363		1.9 (1.7, 2.1)		89.8
26 weeks gestation		353		2.2 (2.1, 2.4)		90.4
Birth		330		2.0 (1.9, 2.2)		87.6
Mean prenatal		365		2.4 (2.3, 2.6)		100
aLOD = 0.4 μg/L.						

The prenatal creatinine-standardized urinary BPA concentrations measured at 16 weeks of gestation, 26 weeks of gestation, and birth were only weakly correlated (Pearson’s correlation coefficients of 0.07, 0.12, and 0.13 for the three pairs of measurements). In bivariate analysis, the OR for a one-unit change in log-transformed mean prenatal BPA concentration was 1.16 (95% CI: 0.90, 1.49). In multivariable analysis, adjusting for season, maternal allergy, mean prenatal serum cotinine concentration, time (categorical), and intervention group, log-transformed mean prenatal urinary BPA concentration was not associated with wheeze [adjusted OR (AOR) = 1.13; 95% CI: 0.86, 1.47].

There was a borderline interaction of mean prenatal urinary BPA concentration with time in the multivariable analysis (*p* = 0.06). We dichotomized prenatal BPA at the median (≥ 2.197 vs. < 2.197 μg/g creatinine) to permit exploration of the interaction of prenatal BPA concentration with time (Table 3). The interaction of dichotomized BPA and time was significant (*p* = 0.002). At 6 months of age, the adjusted odds of wheeze for children born to mothers with a BPA concentration above the median compared with those born to mothers with a BPA concentration below the median was 2.27 (95% CI: 1.28, 4.06); by 3 years, there was no longer a significant association of mean prenatal BPA concentration with wheeze (AOR = 0.57; 95% CI: 0.29, 1.10). There was no statistically significant interaction (*p* > 0.05) of BPA urinary concentrations with any of the other covariates or with sex.

**Table 3:** Adjusted association of mean prenatal BPA urinary concentration (dichotomized at the median)a with child wheeze.

Children reporting wheeze [n (%)]		
Child age	High-BPA group	Low-BPA group	AOR (95% CI)
6 months		46 (25.6)		22 (12.3)		2.27 (1.28, 4.06)
12 months		45 (27.3)		36 (20.6)		1.49 (0.89, 2.48)
18 months		38 (24.4)		31 (19.0)		1.20 (0.69, 2.09)
24 months		22 (15.4)		32 (22.5)		0.55 (0.31, 0.99)
30 months		25 (17.2)		18 (13.2)		1.23 (0.63, 2.40)
36 months		18 (13.4)		26 (20.3)		0.57 (0.29, 1.10)
aMean prenatal BPA urinary concentration was dichotomized at the median (high BPA, ≥ 2.197 μg/g creatinine; low BPA < 2.197 μg/g creatinine).						

*Windows of vulnerability (timing).* In secondary analyses, we used separate multivariable models to estimate associations of wheeze (from birth to 3 years of age) with creatinine-standardized log-transformed maternal urinary BPA concentration at each time point during pregnancy. A one-unit change in log-transformed prenatal urinary BPA concentration at 16 weeks of gestation had a borderline association with wheeze (AOR = 1.21; 95% CI: 0.99, 1.49); wheeze was not associated with BPA at 26 weeks of gestation (AOR = 1.08; 95% CI: 0.87, 1.34) or at birth (AOR = 0.91; 95% CI: 0.73, 1. 15). Interactions of BPA with time (study time point) were not statistically significant in these analyses.

We also explored whether adjusting, rather than standardizing, for urinary creatinine altered our findings, but the results were similar. Specifically, wheeze was associated with a one-unit increase in log-transformed prenatal urinary BPA concentration at 16 weeks of gestation (AOR = 1.24; 95% CI: 1.00, 1.52) but not at 26 weeks of gestation (AOR = 1.07; 95% CI: 0.86, 1.33) or at birth (AOR = 0.89; 95% CI: 0.71, 1. 12).

## Discussion

We evaluated the relationship of prenatal BPA exposure, estimated by measuring maternal urinary BPA concentration, with childhood wheeze in a prospective birth cohort. BPA was detected in the urine of virtually all of the pregnant women. We found that the mean urinary BPA concentration was associated with wheeze in children at 6 months of age, but there was no evidence of a persistent positive association by 3 years of age. In secondary analyses, we explored windows of vulnerability during pregnancy and found an association of wheeze with BPA exposure only during early pregnancy.

Previous investigators have noted an association of exposure to plastic wall materials with the development of bronchial obstruction, adult asthma, and respiratory symptoms typical of childhood asthma (Jaakkola et al. 1999, 2000, 2006; Kim et al. 2007). These studies focused on plastic or plasticizers as a broad category rather than evaluating specific chemicals in plastic. BPA, a chemical found in some plastics, promotes the development of experimental asthma in mouse pups, and perinatal BPA exposure enhanced allergic sensitization, airway hyperresponsiveness, and bronchial eosinophilic inflammation in mice (Midoro-Horiuti et al. 2010). Similarly, we found that prenatal exposure to BPA was associated with wheeze in infants.

Nearly all the children in this study were born to mothers exposed to BPA during pregnancy, based on urinary BPA concentrations. This finding is consistent with NHANES data: Urinary BPA was detected in nearly 93% of participants > 6 years of age (Calafat et al. 2008). The geometric mean urinary BPA concentration among women in the HOME Study cohort was 2.4 μg/g creatinine, compared with 2.6 μg/g creatinine reported in NHANES for 2003–2004 (Calafat et al. 2008).

There are limited human data about the bioavailability and metabolism of BPA during pregnancy. It has been demonstrated that BPA at low levels can cross the human placenta in an *ex vivo* model (Balakrishnan et al. 2010). It has also been noted that BPA levels in human amniotic fluid appear to be higher in early pregnancy than in later pregnancy (Ikezuki et al. 2002). Unfortunately, we did not have direct measures of fetal or amniotic BPA concentrations in this study.

We did not find an association between mean prenatal urinary BPA concentration and wheeze over the entire study period but did find evidence of an association with wheeze in infancy that decreased over time. This early association may be spurious, or it may be analogous to the transient early wheeze phenotype of pediatric asthma (Martinez et al. 1995).

Our primary measure of prenatal BPA exposure was the mean of up to three serial urinary BPA concentrations standardized for creatinine at each time point, but we also examined windows of vulnerability. Increased urinary BPA concentration at 16 weeks of gestation had a borderline association with increased odds of wheeze during the entire time period from 6 months to 3 years of age. This finding suggests that exposure to BPA in early pregnancy might be driving the observed association of mean prenatal BPA concentration and wheeze. There are no other human respiratory studies to compare with this result, but it is consistent with an evaluation of prenatal BPA exposure and child behavior in the same prospective cohort that found that 16-week-gestation urinary BPA concentration was significantly associated with externalizing behaviors (Braun et al. 2009).

We found that BPA concentrations from the three prenatal urinary measurements were weakly correlated. Other biological markers such as serum cotinine measured at similar intervals during pregnancy demonstrate higher correlations (correlation coefficients *r* ≥ 0.8) (Spanier et al. 2011). Despite this weak correlation some investigators have suggested that a single urinary measure of BPA is predictive of long-term exposure (Mahalingaiah et al. 2008). Other investigators have noted that BPA concentrations for an individual vary both within and among days (Ye et al. 2011), and some suggest that studies may need to measure BPA concentration more than once during pregnancy (Braun et al. 2011). We chose to use mean prenatal urinary BPA concentration as the primary exposure measure to represent exposure averaged over pregnancy. However, using the mean prohibited us from adjusting for time-specific urinary creatinine, which was suggested by Barr et al. (2005). Rather than adjusting for urinary creatinine in our analyses, we standardized each BPA concentration before calculating the mean to account for differences in urinary concentration and kidney filtration. Our use of the mean of the maternal urinary BPA concentrations was a conservative approach that could have underestimated associations because it averaged several measures. Future work should evaluate the best approach for BPA exposure assessment throughout pregnancy.

There are several strengths and limitations to this study. First, although having serial measures of BPA is a strength, it forced us to standardize urinary BPA concentration for creatinine. Some investigators suggest that it may be better to adjust for creatinine in the analysis rather than standardize each time point. Adjustment requires inclusion of the creatinine and BPA as separate variables in the analysis. In this case we have three maternal measures, and it would not have been appropriate to place the three maternal BPA concentrations and the three creatinine concentrations in the same analysis. Second, because of the variability of urinary BPA concentrations over time, using this biomarker could result in exposure misclassification. Third, our outcome variable, wheeze, was based on parent report and could have been under- or overreported. Fourth, although the HOME Study is a representative sample of the population in the Cincinnati metropolitan area, it is not a random sample. Last, there was differential attrition for our cohort; the attrition rate for minority, low-income families was higher than for white, affluent families. Future studies should explore the association of BPA exposure with wheeze beyond 3 years of age, with objective measures of lung function, and with the diagnosis of asthma.

## Conclusion

We found that mean prenatal BPA exposure, assessed from maternal urinary BPA concentration, was associated with increased odds of wheeze at 6 months of age, but the association diminished by 3 years of age. Evaluating BPA exposure at each prenatal measurement time demonstrated an association of wheeze with BPA at 16 weeks of gestation but not at 26 weeks of gestation or at birth, which suggests that early pregnancy may be the most sensitive time window of exposure. In the analyses of each separate prenatal measurement, there was no evidence that the effect diminished across the 3 years. If exposure to BPA is confirmed to increase the risk for developing wheeze or asthma, it may help explain the rising rates of childhood asthma and would have important policy implications for women of childbearing age and their children.
